# The *cyl* Genes Reveal the Biosynthetic and Evolutionary Origins of the Group B *Streptococcus* Hemolytic Lipid, Granadaene

**DOI:** 10.3389/fmicb.2019.03123

**Published:** 2020-01-21

**Authors:** Blair Armistead, Christopher Whidbey, Lakshminarayan M. Iyer, Pilar Herrero-Foncubierta, Phoenicia Quach, Ali Haidour, L. Aravind, Juan Manuel Cuerva, Heather B. Jaspan, Lakshmi Rajagopal

**Affiliations:** ^1^Department of Global Health, University of Washington, Seattle, WA, United States; ^2^Center for Global Infectious Disease Research, Seattle Children’s Research Institute, Seattle, WA, United States; ^3^Computational Biology Branch, National Center for Biotechnology Information, National Institutes of Health, Bethesda, MD, United States; ^4^Department of Organic Chemistry, University of Granada, Granada, Spain; ^5^Department of Pediatrics, University of Washington School of Medicine, Seattle, WA, United States; ^6^Department of Pathology, Institute of Infectious Disease and Molecular Medicine, University of Cape Town, Cape Town, South Africa

**Keywords:** Group B *Streptococcus*, bacterial toxin, microbial evolution, virulence factor, Gram-positive bacteria

## Abstract

Group B *Streptococcus* (GBS) is a β-hemolytic, Gram-positive bacterium that commonly colonizes the female lower genital tract and is associated with fetal injury, preterm birth, spontaneous abortion, and neonatal infections. A major factor promoting GBS virulence is the β-hemolysin/cytolysin, which is cytotoxic to several host cells. We recently showed that the ornithine rhamnolipid pigment, Granadaene, produced by the gene products of the *cyl* operon, is hemolytic. Here, we demonstrate that heterologous expression of the GBS *cyl* operon conferred hemolysis, pigmentation, and cytoxicity to *Lactococcus lactis*, a model non-hemolytic Gram-positive bacterium. Similarly, pigment purified from *L. lactis* is hemolytic, cytolytic, and identical in structure to Granadaene extracted from GBS, indicating the *cyl* operon is sufficient for Granadaene production in a heterologous host. Using a systematic survey of phyletic patterns and contextual associations of the *cyl* genes, we identify homologs of the *cyl* operon in physiologically diverse Gram-positive bacteria and propose undescribed functions of *cyl* gene products. Together, these findings bring greater understanding to the biosynthesis and evolutionary foundations of a key GBS virulence factor and suggest that such potentially toxic lipids may be encoded by other bacteria.

## Introduction

Hemolytic activity is a key determinant of colonization and pathogenesis in Group B *Streptococcus* (GBS) (Nizet et al., 1996; Doran et al., 2002, 2003; Patras et al., 2013; Whidbey et al., 2013, 2015b; Boldenow et al., 2016), a Gram-positive bacterium that resides in the lower genital and/or gastrointestinal tract of approximately 18% of women and is a major cause of preterm birth and severe neonatal infections (Russell et al., 2017a, b; Seale et al., 2017a, b). Nearly 100 years ago, GBS isolates of human origin were first described as β-hemolytic (Brown, 1920; Ayers and Philip, 1922), which was invariantly linked to a pigmented phenotype (Nizet et al., 1996; Spellerberg et al., 2000; Pritzlaff et al., 2001). Recently, the GBS hemolysin and pigment were shown to be one and the same (Whidbey et al., 2013), initiating a shift in understanding this virulence factor. The GBS hemolytic pigment, also known as Granadaene (Rosa-Fraile et al., 2006), is a cell surface-associated (Platt, 1995) ornithine rhamnolipid consisting of a 12-alkene chain and is unrelated to other commonly studied Gram-positive pore-forming toxins, such as lysteriolysin O (Hamon et al., 2012) or alpha toxin (Seilie and Bubeck Wardenburg, 2017), which are proteinaceous in nature. Many gaps remain in our understanding of Granadaene, including the specifics of its biosynthesis in bacterial cells and reasons why GBS may have evolved to produce this potent toxin. Such insights would improve our understanding of this critical GBS virulence factor as well that of other similar, potentially toxic microbial lipids.

The *cyl* operon (*cylX-K*) is necessary for hemolytic pigment production in GBS (Spellerberg et al., 1999, 2000; Pritzlaff et al., 2001; Whidbey et al., 2013). We have previously postulated that enzymes encoded in the *cyl* operon use acetyl-CoA, malonyl Co-A, ornithine, and rhamnose as the building blocks for pigment biosynthesis in GBS (Whidbey et al., 2013). Here, for the first time, we show that heterologous expression of the GBS *cyl* operon is sufficient to confer the production of functional Granadaene in *Lactococcus lactis*, a generally recognized as safe (GRAS) Gram-positive bacterial strain (Cavanagh et al., 2015). Phyletic analysis revealed previously undescribed functional categories of *cyl* gene products, indicated that the *cyl* operon genes are present in a diverse range of Gram-positive bacteria, and suggested that pigment biosynthesis evolved in free-living bacteria, likely as a photoprotectant or as a defense mechanism against competing organisms. Collectively, these findings provide biosynthetic and evolutionary insight into a critical GBS virulence factor.

## Results and Discussion

### Heterologous Expression of the GBS *cyl* Operon in *Lactococcus lactis* Confers Hemolysis, Pigmentation, and Cytotoxicity

**FIGURE 1:**
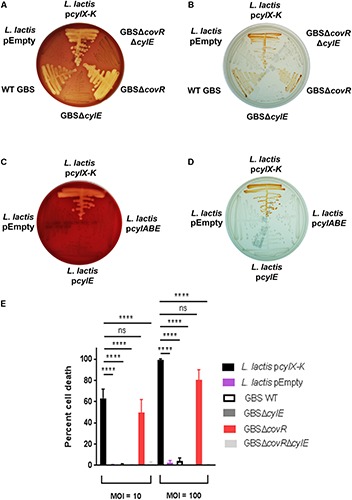
Complementation of *L. lactis* with pcylX-K, but not empty plasmid vector, pEmpty, conferred hemolysis **(A)** and pigmentation **(B)** similar to that observed for hemolytic GBS, including strains that lack the transcriptional repressor of the *cyl* operon, CovR (GBSΔ*covR*). Of note, GBS strains lacking CylE (i.e., GBSΔ*cylE*, GBSΔ*covR*Δ*cylE*) are neither hemolytic nor pigmented. Complementation of *L. lactis* with *cylE* alone or *cylA*, *cylB*, and *cylE* did not confer hemolysis **(C)** or pigmentation **(D)** to *L. lactis*. **(E)** *L. lactis* p*cylX-K, L. lactis* pEmpty, GBS WT, GBSΔ*cylE*, GBSΔ*covR*, or GBSΔ*covR*Δ*cylE* were incubated with human neutrophils for 4 h at an MOI of 10 and 100, and neutrophil death was measured by LDH release into cell supernatants relative to Triton X-100 and PBS-only controls. Mean and standard error from three independent experiments performed in triplicate are shown. One-way ANOVA with Tukey’s post-test was performed. ^∗∗∗∗^ indicates *p* < 0.0001, and ns indicates not significant, or *p* ≥ 0.05.

Our recent studies (Whidbey et al., 2013, 2015b; Gendrin et al., 2015; Boldenow et al., 2016) have revealed that the hemolytic pigment encoded by the GBS *cyl* operon genes is cytotoxic to many eukaryotic cells. Given that GBS colonizes the human host with other commensal microbes, we wondered whether horizontal gene transfer of this operon would enable production of the hemolytic lipid pigment [also known as Granadaene (Rosa-Fraile et al., 2006)] to non-hemolytic bacteria. To test this hypothesis, we expressed the *cyl* operon genes in a model Gram-positive bacterium, *Lactococcus lactis*. First, we cloned the 12-gene *cyl* operon (i.e., genes *cylX-cylK*) into a complementation vector pDC123 (Chaffin and Rubens, 1998) to generate the p*cylX-K* plasmid. Then, we complemented a non-hemolytic/non-pigmented GBS strain lacking the entire *cyl* operon [GBSΔ*cylX-K* (Whidbey et al., 2013)] with the p*cylX-K* plasmid and confirmed that the plasmid restored hemolysis and pigmentation as observed on blood agar and Granada media (Supplementary Figure S1). Next, we transformed the p*cylX-K* plasmid into *L. lactis* to generate *L. lactis* p*cylX-K*. As a control, *L. lactis* was transformed with the empty plasmid vector (*L. lactis* pEmpty). Like hemolytic GBS, β-hemolysis and pigmentation were observed with *L. lactis* p*cylX-K* and not *L. lactis* pEmpty (Figures 1A,B). Consistent with previous observations with GBS (Whidbey et al., 2013), plasmids encoding only *cylE* (p*cylE*) or *cylABE* (p*cylABE*) were insufficient to confer hemolysis and pigmentation to *L. lactis* (Figures 1C,D). Also, the recombinant p*cylX-K* plasmid did not confer hemolysis to *E. coli* (Supplementary Figure S2), which we predict is due to the abundance of rare codons present in the *cyl* operon (Supplementary Table S1). Together, these results indicate that expression of the *cyl* operon is necessary and sufficient for hemolysis and pigmentation in GBS and even in *L. lactis*.

We next tested if the *cyl* operon expression by *L. lactis* conferred neutrophil cytotoxicity. In GBS, expression of the *cyl* operon is transcriptionally repressed by the CovR/S two-component system, and the absence of the repressor CovR results in increased expression of the hemolytic pigment, Granadaene (Lamy et al., 2004; Rajagopal et al., 2006; Lembo et al., 2010). GBS strains lacking a functional CovR (Δ*covR*) cause significantly greater cytotoxicity in neutrophils compared to wildtype (WT) GBS and isogenic non-pigmented/non-hemolytic mutants due to enhanced production of Granadaene (Lamy et al., 2004; Jiang et al., 2005; Rajagopal et al., 2006; Boldenow et al., 2016). Furthermore, GBS strains lacking CovR have been identified and isolated from women in preterm labor (Whidbey et al., 2013) and from patients with other GBS infectious morbidities (Sendi et al., 2009; Lupo et al., 2014; Almeida et al., 2015, 2017; Whidbey et al., 2015a). We hypothesized that *L. lactis* p*cylX-K*, which lacks transcriptional repressors specific to the *cyl* operon, would induce neutrophil cytotoxicity similar to GBSΔ*covR*. To test this, we exposed primary human neutrophils to *L. lactis* p*cylX-K* at a multiplicity of infection (MOI) of 10 or 100 and measured cytotoxicity by lactate dehydrogenase (LDH) release in cell supernatants as described (Whidbey et al., 2013; Gendrin et al., 2015; Boldenow et al., 2016). WT GBS or hyper-hemolytic/pigmented GBSΔ*covR* were also included for comparison. As controls, we exposed neutrophils to *L. lactis* pEmpty, non-pigmented/non-hemolytic GBSΔ*cylE*, and non-pigmented/non-hemolytic GBSΔ*covR*Δ*cylE*. The results, shown in Figure 1E, demonstrate that the cell death caused by *L. lactis* p*cylX-K* was similar to that caused by the hyper-hemolytic strain GBSΔ*covR*. Importantly, cytotoxicity in cells exposed to *L. lactis* p*cylX-K* was greater than those exposed to *L. lactis* pEmpty. These results demonstrate that expression of the *cyl* operon in *L. lactis* is sufficient to confer neutrophil cytotoxicity like hyper-hemolytic/hyper-pigmented GBS.

### Pigment Isolated From *L. lactis* p*cylX-K* Is Identical to Granadaene From GBS

**FIGURE 2:**
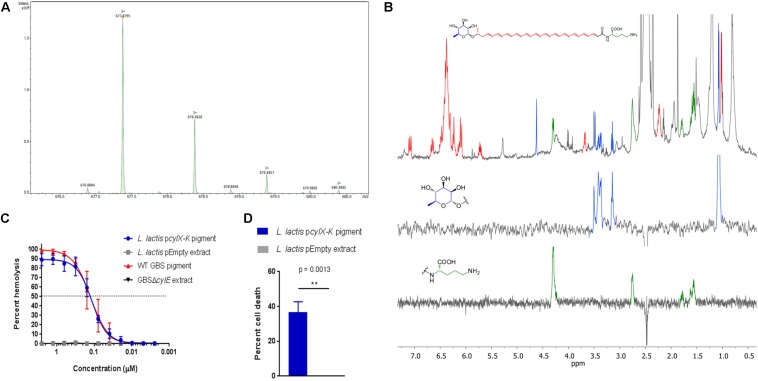
Pigment extracted and purified from *L. lactis* p*cylX-K* is identical to Granadaene extracted from WT GBS. **(A)** High resolution mass spectrometry was performed on pigment extracted from *L. lactis* p*cylX-K* and demonstrated an M + H ion at *m/z* 677.3795, which is associated with an ion formula of C_39_H_53_N_2_O_8_ (expected mass of 677.3796). **(B)** ^1^H NMR analyses on *L. lactis* p*cylX-K* pigment revealed a signal corresponding to a polyene structure (7.20–5.5 ppm), as well as signals corresponding to an ornithine (4.32, 2.77, 1.80, 1.59, and 1.64 ppm) and a rhamnose (4.63, 3.52, 3.43, 3.39, 3.16, and 1.09 ppm). **(C)** HPLC-purified pigment from *L. lactis* p*cylX-K* or WT GBS in DMSO + trifluoroacetic acid + starch (DTS) was added to human erythrocytes in twofold serial dilutions starting from 2.5 to 0.0024 μM for 1 h. As controls, equivalent amounts of extracts from *L. lactis* pEmpty and from non-pigmented/hemolytic GBSΔ*cylE* were tested. Mean and standard error from three independent experiments performed in triplicate are shown. The EC_50_ for *L. lactis* p*cylX-K* pigment (0.128 μM, 95% CI: 0.108, 0.153) is no different than that of WT GBS pigment/Granadaene (0.127 μM, 95% CI: 0.100, 0.162). **(D)** Primary human neutrophils were incubated with 0.5 μM purified pigment from *L. lactis* p*cylX-K* for 4 h at 37°C. As a control, neutrophils were treated with an equivalent amount of *L. lactis* pEmpty extract. Neutrophil death was measured by LDH release into cell supernatants relative to Triton X-100 and PBS-only controls. Mean and standard error from three independent experiments performed in triplicate are shown. An unpaired *t*-test was performed, and *p* = 0.0013. ^∗∗^ indicates that *p* < 0.01.

We next tested if pigment produced by *L. lactis* p*cylX-K* was similar in structure to the GBS pigment, Granadaene. Accordingly, we extracted and purified pigment from *L. lactis* p*cylX-K* using methods described with GBS (Rosa-Fraile et al., 2006; Whidbey et al., 2013, 2015b; Gendrin et al., 2015; Boldenow et al., 2016) (see section “Materials and Methods”). As a control, we performed the same extraction and purification procedure on *L. lactis* pEmpty. In mass spectrometry studies, pigment purified from *L. lactis* p*cylX-K* demonstrated an M + H ion at *m/z* 677.3795 (Figure 2A). This exact mass is associated with an ion formula of C_39_H_53_N_2_O_8_ (expected mass of 677.3796), which is identical to that of Granadaene from GBS, as described (Rosa-Fraile et al., 2006; Whidbey et al., 2013). As expected, no peaks corresponding to the mass of Granadaene were observed in the extract from control *L. lactis* pEmpty. To gain further insight into the structure of pigment produced by *L. lactis* p*cylX-K*, we performed ^1^H NMR studies (Figure 2B). These analyses revealed a signal corresponding to a polyene structure (7.20–5.5 ppm), as well as signals corresponding to an ornithine (4.32, 2.77, 1.80, 1.59, and 1.64 ppm) and a rhamnose (4.63, 3.52, 3.43, 3.39, 3.16, and 1.09 ppm), which are identical to those described for natural Granadaene (Rosa-Fraile et al., 2006; Whidbey et al., 2013). The ornithine and rhamnose were also confirmed to be present in *L. lactis* p*cylX-K* pigment extract by 1D TOCSY. Importantly, none of these structures were present in the NMR analysis of *L. lactis* pEmpty extract. Of note, little is known about how Granadaene is tethered to the bacterial cell membrane, and while ornithine is present on the terminal end of extracted Granadaene from GBS and *L. lactis* p*cylX-K*, is it possible that solvents (namely trifluoracetic acid) may alter the terminal moiety during the extraction procedure. Future studies examining the structure of Granadaene embedded in intact bacterial membranes will help discern the nature of Granadaene’s terminal moiety prior to extraction. Nevertheless, our results demonstrate that the nominal formula and the structure of pigment produced by *L. lactis* p*cylX-K* are indistinguishable from those previously assigned to Granadaene (Figure 2B; Rosa-Fraile et al., 2006; Whidbey et al., 2013).

To confirm that pigment purified from *L. lactis* p*cylX-K* possessed hemolytic and cytolytic activity analogous to Granadaene, we performed a hemolytic titer assay using the *L. lactis* p*cylX-K* pigment extract (Nizet et al., 1996; Whidbey et al., 2013). The results shown in Figure 2C indicate that purified pigment from *L. lactis* p*cylX-K* possessed hemolytic activity similar to that of Granadaene (Whidbey et al., 2013). Moreover, the EC_50_ (the effective concentration at which 50% of human red blood cells are lysed) of *L. lactis* p*cylX-K* pigment (0.128 μM, 95% CI: 0.108, 0.153) was no different than that of Granadaene (0.127 μM, 95% CI: 0.100, 0.162).

Finally, we sought to determine if *L. lactis pcylX-K* pigment is cytolytic to host cells like Granadaene. Purified Granadaene was previously reported to be cytolytic to host cells such as neutrophils (Boldenow et al., 2016). Human neutrophils isolated from fresh adult blood were treated with *L. lactis* p*cylX-K* pigment (0.5 μM) or equivalent amount of control extract from *L. lactis* pEmpty for 4 h, as described (Boldenow et al., 2016), and cytotoxicity was measured by LDH release in cell supernatants. As observed previously with Granadaene, *L. lactis* p*cylX-K* pigment caused significantly greater cell death than non-hemolytic control extracts (Figure 2D; Boldenow et al., 2016). Together, these data indicate that purified pigment from *L. lactis* p*cylX-K* possesses hemolytic and cytolytic activity that are no different than Granadaene. Furthermore, our findings confirm that the *cyl* operon encode proteins sufficient for production of this ornithine rhamnolipid in a heterologous Gram-positive bacterium.

### Phyletic Analysis Suggests the *cyl* Operon Evolved Prior to the Diversification of Gram-Positive Bacteria

**FIGURE 3:**
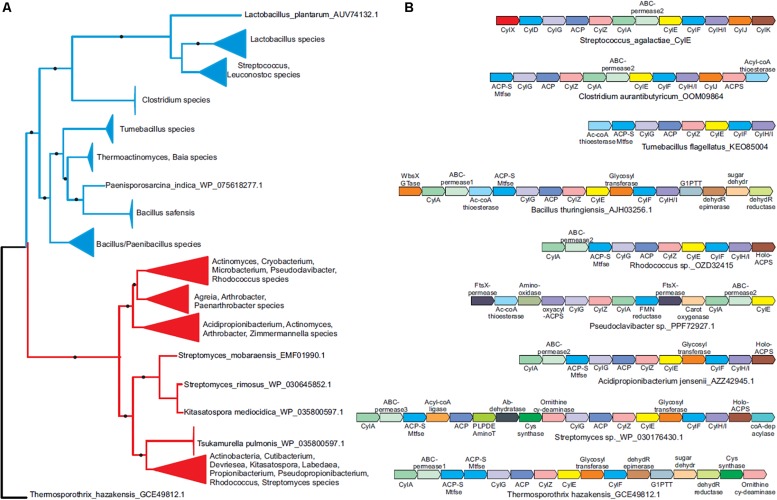
Phyletic analysis suggests the *cyl* operon evolved prior to the diversification of Gram-positive bacteria. **(A)** A phylogenetic tree of the CylE protein is shown. Clades with strong bootstrap support were collapsed into filled triangles for convenience (see Supplementary Figure S3 for full tree). Firmicute and actinobacterial branches are colored blue and red, respectively. **(B)** Representative *cyl* operons derived from the phylogenetic tree in **(A)** are shown. Genes are shown as boxed arrows, with the arrow head pointing to the 3′ gene. Operons are labeled using the accession number of the *cylE* gene in the operon. Proteins are denoted using their species names followed by their Genbank accession number or gene names.

Our heterologous expression experiments suggest a possible advantage for lateral transfer of *cyl* genes between various bacteria. To understand the evolutionary origins of the ornithine rhamnolipid pigment, we conducted a systematic survey of the phyletic patterns and contextual associations of the *cyl* genes. We found that *cyl* genes are present in diverse Gram-positive phyla such as firmicutes and actinobacteria as well as the chloroflexi *Thermosporothrix*. A phylogenetic tree using the *cylE* acyl transferase gene, which is universally present in *cyl* operons, showed that the actinobacterial and firmicute proteins group within their respective bacterial clades, whereas the *Thermosporothrix cylE* groups outside the two principal clades (Figure 3A and Supplementary Figure S3). The *Thermosporothrix* operons seem to contain elements of both the actinobacterial and firmicute types of operon, supporting that it has retained features of an ancestral version (Figure 3A and Supplementary Figure S3). The presence of *cyl* operons in diverse firmicutes (spore formers and non-spore formers) and actinobacteria, and a largely vertical inheritance of the firmicute and actinobacterial versions, indicates that the pigment biosynthesis genes evolved prior to the diversification of Gram-positive bacteria.

We identified five functional groups of genes in homologous *cyl* operons (Table 1): (1) those involved in lipid biosynthesis, (2) ornithine biosynthesis, (3) acyl-lipid transfer to the ornithine, (4) sugar biosynthesis and transfer, and (5) pigment export. Of these, our data indicate a vertically inherited orthologous core of seven genes comprising of *cylA*, *cylG*, *acpC*, *cylZ*, *cylE*, *cylF*, and *cylH*. This likely comprises the most ancient core of the operon. Further, non-orthologous but biochemically functionally equivalent versions of four genes are observed, which encode the following: (1) CylK (*Streptococcus, Lactococcus, Leuconostoc*) and ACP synthase (actinobacteria, *Clostridium* and *Paenisporosarcina* species); (2) CylJ (Streptococci, certain *Lactococci* and *Clostridium* species) and one or two distinct glycosyltransferases (various firmicutes, actinobacteria and chloroflexi) involved in sugar conjugation; (3) CylD (Streptococci, *Leuconostoc* and *Lactococci*) and ACP-S-malonyltransferase (in various firmicutes, chloroflexi and actinobacteria); and 4) at least three distinct versions of ABC-transporter permease subunits have been alternatively recruited for pigment transport (Figure 3B and Table 1). Of note, the non-orthologous displacements are supported by change in operon position of the genes (Supplementary Figure S4). Taken together, this suggests a core of approximately eleven genes in an ancient operon prior to the divergence of actinobacteria and firmicutes, which encoded proteins belonging to one of the five functional groups involved in pigment synthesis (Table 1).

**TABLE 1:** Genes in *cyl* operons encode proteins that fall into one of five functional categories.

			**Orthologs**	**Non-orthologous,**
			**conserved**	**functionally equivalent**
**Functional**	**Gene product in**		**in all *cyl***	**gene products in other**
**category**	**GBS *cyl* operon**	**Putative function (reference)**	**operons?**	***cyl* operons**
Lipid biosynthesis	CylX	Generates malonyl- CoA units (Whidbey et al., 2013)	Yes	
	CylK	Phosphopantetheinyl transferase, involved in ACP synthesis (Whidbey et al., 2013)	No	ACP synthase
	ACP	Acyl carrier protein (Spellerberg et al., 1999)	Yes	
	CylD	Conjugates ACP to malonyl-CoA units (Spellerberg et al., 1999)	No	ACP-s-malonyltransferase
	CylH/I	Forms initial fatty acid-ACP complex and adds new keto groups to elongate unsaturated fatty acid chain (Pritzlaff et al., 2001; Whidbey et al., 2013)	Yes	
	CylG	Reduces keto group added by CylH/I to hydroxyl (Spellerberg et al., 1999; Whidbey et al., 2013)	Yes	
	CylZ	Further reduces keto group to alkene (Whidbey et al., 2013)	Yes	
Ornithine biosynthesis	CylF	Generates ammonia for ornithine biosynthesis (this study)	Yes	
Acetyltransferase	CylE	Replaces ornithine with ACP group on unsaturated fatty acid (Whidbey et al., 2013)	Yes	
Sugar biosynthesis and conjugation	CylJ	Attaches glycosyl group to unsaturated fatty acid (Forquin et al., 2007)	No	glycosyltransferase, WbsX glucosyltransferase
Pigment export	CylA	Transport of pigment to cell surface; ATP binding domain (Spellerberg et al., 1999; Gottschalk et al., 2006)	Yes	
	CylB (ABC permease 2)	Transport of pigment to cell surface; transmembrane domain (Spellerberg et al., 1999; Gottschalk et al., 2006)	No	ABC permease1, ABC permease3

*Although orthologs of all genes present in the GBS *cyl* operon are not present in other *cyl* operons, non-orthologous, functionally equivalent proteins are encoded. A biosynthetic pathway for GBS pigment was previously proposed (Whidbey et al., 2013). Briefly, CylX-generated malonyl-CoA units are linked to the acyl carrier protein (ACP) by CylD. The fatty-acid like pathway is initiated through the action of CylI, which joins the malonyl-CoA to an initial fatty acid-ACP complex. The fatty acid intermediate is reduced by CylG and CylZ, creating an alkene. CylI then utilizes the alkene as a substrate and adds another keto group, which is reduced further, resulting in another alkene on the unsaturated fatty acid chain. The elongation/reduction cycle repeats until the polyene chain contains twelve alkenes. Meanwhile, CylF cleaves amino acids such as glycine to generate ammonia, which can then be utilized by ornithine cyclodeaminase (OCD) or another functionally similar enzyme to generate ornithine. CylE replaces ACP with ornithine in the full-length polyene chain, and CylJ links the terminal glycosyl group to the polyene chain in the final step of pigment biosynthesis. The pigment is then transported to the bacterial cell surface via the CylA/B ABC transporter.*

We observe that some *cyl* genes are exclusive to specific bacterial lineages, suggesting that they were acquired post-divergence. For instance, genes encoding CylJ and CylD are likely to have displaced those encoding the ancestral glycosyltransferase and ACP-S-malonyltransferase, respectively, in certain Streptococci (Figure 3B). These also include rhamnose biosynthesis genes, which are only seen in firmicutes and chloroflexi, as well as ornithine cyclodeaminase (OCD), PLP-dependent aminotransferases, acyl coA ligase and various redox enzymes, which are only found in actinobacteria and partly in chloroflexi. We predict that these genes evolved to increase the availability of the building blocks for pigment biosynthesis or add diversity to the functional groups on the pigment. For example, the presence of two glycosyltransferases in various Bacillus species in the *cyl* operons (Figure 3B and Supplementary Figure S3) could imply that these species possess a more complex sugar modification than rhamnose, and this warrants further study. In short, while there is little evidence to suggest extensive lateral transfer of *cyl* genes, our data indicate that individual genes were likely lost or displaced by non-orthologous genes across various species.

### The Evolution of CylE and CylF Conferred Critical Functions in Granadaene Biosynthesis to the *cyl* Operon

One of the previously unresolved questions was the role of the lipoate-dependent aminomethyltransferase (CylF), as this is a universally conserved gene encoded by *cyl* operons. We predict that this is involved in ornithine biosynthesis. We infer this based on the presence of OCD-encoding genes in some actinobacterial *cyl* operons (Figure 3B and Table 1), which suggests in these organisms and, perhaps more generally in all organisms with the pigment, ornithine is synthesized by combining proline with ammonia. This provides a role for the aminomethylase CylF as this enzyme splits amino acids such as glycine to give rise to ammonia (Kikuchi et al., 2008). Given the need for ammonia in the ornithine synthesis by the OCD enzyme, we predict that CylF generates ammonia in the local milieu of the pigment biosynthesis complex, which is then used by OCD to make ornithine. Of the five basic components of the *cyl* operon, the fatty acid biosynthesis genes often appear together in other contexts. However, the accretion of the acetyltransferase CylE and CylF in an ancient bacterium were perhaps the key events in the fixation of the *cyl* genes for pigment biosynthesis.

Given the phyletic patterns, we hypothesize that the ancient *cyl* operon evolved in free-living bacteria, where we conceive possible biological roles that might not be mutually exclusive. First, the ornithine rhamnolipid might be involved in photoprotection and/or membrane stabilization, as with many microbial pigments (Taylor, 1984; Howardwilliams et al., 1989; Rajagopal et al., 1997; Slouf et al., 2012; Madden et al., 2019). Second, it might have evolved as a general defense against competing organisms in which the pigment disrupts their cell membrane. The latter is supported by the hemolytic and cytolytic activity of the Granadaene pigment in GBS (Whidbey et al., 2013, 2015b; Boldenow et al., 2016) and *cyl*-expressing *L. lactis* (Figures 1, 2). The presence of an identical or similar hemolytic pigment in a range of Gram-positive bacteria with *cyl* genes (Vanberg et al., 2007; Deptula et al., 2019), as well as our heterologous expression experiments, suggest that Granadaene can potentially function across a physiologically diverse group of organisms if they likely possess a Gram-positive-type cell wall structure. In fact, a recent study correlated pigmentation with hemolysis and *cyl* gene expression in *Acidipropionibacterium* species that have roles in cheese production and food preservation (Deptula et al., 2019). Accordingly, the hemolytic and cytotoxic properties of other pigmented, *cyl* operon-containing microbes found in soil environments or utilized for industrial application warrant further examination.

These findings broaden our understanding of the biosynthesis and evolutionary history of Granadaene in GBS, along with that of other homologous microbial lipid pigments. Although our phyletic analyses do not establish horizontal gene transfer of the entire *cyl* operon, our heterologous gene expression studies indicate that this remains a formal possibility, particularly in Gram-positive bacteria. Because GBS cohabitates the human lower genital and gastrointestinal tract with other commensal bacteria, the possibility of lateral transfer of the *cyl* operon to otherwise avirulent bacteria in the human niche should be explored further.

## Materials and Methods

### Ethics Statement

Written informed consent for donation of human blood was obtained from patients, per the Principles in the WMA Declaration of Helsinki and Department of Health and Human Services Belmont Report. The study was approved by the Seattle Children’s Research Institute Institutional Review Board (protocol #11117). Children under the age of 18 were not recruited for donation of human blood.

### Bacterial Strains

The GBS strain A909 was used in all studies. A909 is a clinical isolate obtained from an infected human neonate and is classified as serotype Ia (Lancefield et al., 1975). The Δ*cylE*, Δ*covR*, and Δ*covR*Δ*cylE* mutants were derived from A909 as described (Rajagopal et al., 2006; Lembo et al., 2010). Cultures of GBS were grown in tryptic soy broth (TSB; Difco Laboratories) at 37°C in 5% CO_2_, and cultures of *Escherichia coli* MC1016 were grown in lysogeny broth (LB; Difco Laboratories) at 37°C. Cultures of *Lactococcus lactis* were grown in TSB at 30°C in 5% CO_2_. Culture growth for all bacterial strains was measured at 600 nm, and bacterial strains were washed twice in PBS before being used in experiments. Photographs of bacterial strains on blood agar (Remel) or Granada medium (Hardy Diagnostics) were captured with an SLR camera (EOS Rebel XSi 12.2MP; Cannon) with an 18–55 mm zoom lens and processed using Photoshop CC (Adobe).

### Heterologous Expression of the *cyl* Operon in *Lactococcus lactis*

The *cyl* operon was amplified using high fidelity PCR and primer pairs cyl1, cyl2, and cyl3 (IDT, see Supplementary Table S2) to obtain three overlapping DNA fragments. The PCR fragments were ligated to a PCR fragment representing the vector pDC123 (Chaffin and Rubens, 1998), referred to in the main text and hereafter as pEmpty) using Gibson Assembly. The recombinant plasmid, which contained a chloramphenicol resistance gene, was electroporated into *Escherichia coli* MC1061 and then isolated by midiprep (Qiagen). After the presence of the *cyl* operon within the recombinant plasmid was confirmed by PCR amplification, the recombinant plasmid was transformed into electrocompetent *Lactococcus lactis*. Upon the addition of the recombinant or empty plasmid, *L. lactis* was grown in the presence of 5 μg/mL chloramphenicol (Sigma-Aldrich).

### Isolation and Purification of Pigment From GBS and *L. lactis*

Pigment was isolated from wildtype GBS and *L. lactis* p*cylX-K* as described, with minor modifications (Rosa-Fraile et al., 2006; Whidbey et al., 2013, 2015b; Gendrin et al., 2015; Boldenow et al., 2016). Wildtype GBS A909 or *L. lactis* p*cylX-K* were grown in 500 mL Granada medium (De La Rosa et al., 1992), and the bacterial pellet was washed twice with HPLC-grade water and twice with DMSO. Then, the pellet was resuspended in DMSO + 0.1% trifluoroacetic acid (TFA; Sigma-Aldrich) to extract the pigment. Extractions with DMSO + 0.1% TFA continued until no pigmentation was observed in the bacterial pellet. The pigment extract was pooled and precipitated overnight with NH_4_OH (Scientific Products). The precipitated pellet was washed twice with HPLC-grade water, and twice with DMSO. Pigment was then extracted using DMSO + 0.1% TFA as above, pooled, and then column-purified using high pressure liquid chromatography (HPLC, Shimadzu 10A system) with a Vydac 214TP C4 column (solvent: DMSO + 0.1% TFA, flow rate: 1 mL/min; detection: 489 nm). Fractions were collected between 10.5 min and 17.5 min of each run (when detection level was >100,000 μV, see Supplementary Figure S5). Purified fractions were pooled, precipitated with NH_4_OH (Scientific Products), washed three times with HPLC-grade water and then twice with DMSO, and lyophilized to dryness. Lyophilized pigment toxin was stored at −80°C, and working pigment toxin solutions were dissolved in DMSO + 0.1% TFA + 20% starch (DTS), as described (Whidbey et al., 2013). The purification and isolation procedure was also performed on corresponding GBS A909Δ*cylE* (non-pigmented/non-hemolytic isogenic mutant of wildtype A909) and *L. lactis* pEmpty (transformed with empty vector, non-pigmented/non-hemolytic) as described above. Extract from A909Δ*cylE* and *L. lactis* pEmpty were used as controls for pigment in all experiments, along with the DTS solvent. For NMR analysis, purified pigment from *L. lactis* p*cylX-K* or control extract from *L. lactis* pEmpty were resuspended in DMSO + 0.1% TFA. ^1^H, ^13^C, ^1^H-COSY NMR experiments were performed at 298K on a Varian 600 MHz NMR Spectrometer. Residual DMSO-d_5_ was used to calibrate chemical shifts. High resolution mass spectra (HRMS) of samples dilute in DMSO + 0.1% TFA were recorded on a mass spectrometer using via ESI.

### Hemolytic Assays

Lyophilized pigment toxin or control extracts were dissolved in DTS to a final concentration of 200 μM and were incubated overnight at room temperature and protected from light before use. To perform the hemolytic titer, twofold serial dilutions of purified pigment in DTS from A909 WT or *L. lactis* p*cylX-K* were performed in PBS in a final volume of 100 μL. Twofold serial dilutions of control extracts (from GBSΔ*cylE* or *L. lactis* pEmpty) in DTS were also performed in PBS as controls. The samples were incubated with 100 μL of EDTA-treated human red blood cells (0.5% in PBS) in 96-well plates for 1 h at 37°C. Then, the 96-well plate was centrifuged for 4 min at 300 × *g* to pellet the unlysed red blood cells, and supernatants were transferred to a replica 96-well plate. Hemoglobin release was measured by recording the absorbance at 420 nm of the supernatants (Spectramax i3x Plate Reader, Molecular Devices), and percent hemolysis was determined relative to red blood cells treated with positive control Triton X-100 (0.1%, Sigma-Aldrich) and negative control PBS only.

### Isolation of Neutrophils From Adult Human Blood

As described (Boldenow et al., 2016), 5–15 mL of human adult blood was collected from independent healthy human adults into EDTA tubes (BD Bioscience). Immediately following collection, neutrophils were isolated using a MACSxpress neutrophil isolation kit, per the manufacturer’s instructions (Miltenyi Biotec). Cells were then pelleted, and any residual red blood cells (RBC) were removed by re-suspending the cell pellet in RBC lysis solution (0.15 nM NH_4_Cl, 1 mM NaHCO_3_) for 15 min at room temperature. Following RBC lysis, cells were washed with Roswell Park Memorial Institute 1640 tissue culture medium containing L-glutamine (Corning; hereafter referred to as RPMI-G). Neutrophil purity in the prepared cell suspension was assessed by examining the proportion of cells positive for the neutrophil cell markers CD15 (PerCP/Cy5.5, clone HI98, BD Biosciences) and CD16 (FITC, clone 3G8, BD Biosciences) by flow cytometry. Briefly, approximately 1 × 10^6^ cells from the neutrophil purification preparation or 1 × 10^6^ cells from whole blood (following two RBC lysis steps, as described above) were incubated with Fc receptor block (1:200, BD Biosciences) for 15 min at room temperature. Then, immunofluorescent antibodies were added to the cells at concentrations recommended by the manufacturer (1:10, CD15-PerCP/Cy5.5; 1:200, CD16-FITC), and cells incubated for 30 min at room temperature. Stained cells were washed twice in FACS (fluorescence-activated cell sorting) buffer (1 mM EDTA, 25 mM HEPES, 1% BSA (w/v) in PBS) and were analyzed immediately on an LSR II flow cytometer (BD Biosciences). Single-stained flurochrome-reactive AbC beads (Thermo Fisher) and unstained cells were used for compensation. Data were analyzed using FlowJo v. 10.1 (FlowJo, LLC).

### Testing Bacterial Strains and Pigment Extracts for Neutrophil Cytotoxicity

Neutrophils were isolated as described above and were seeded in a U-bottom 96-well plate at approximately 4 × 10^5^ cells/mL in 90 μL RPMI-G. *L. lactis* p*cylX-K* and *L. lactis* pEmpty were grown to mid-exponential growth phase (OD_600_ = 0.3), washed twice in sterile PBS, and added to neutrophils at a multiplicity of infection (MOI) of 10 and 100. MOIs were confirmed by dilution plating. Additionally, pigment in DTS from *L. lactis* pcylX-K or WT GBS was added to neutrophils at a final concentration of 0.5 μM. As positive and negative controls for all assays, neutrophils were incubated in 0.1% Triton X-100 (Sigma Aldrich) or sterile PBS, respectively. After incubating for 4 h at 37°C, cells were analyzed for cytotoxicity by the presence of cytoplasmic lactate dehydrogenase (LDH) in cell supernatants using the colorimetric LDH kit (Clontech), per the manufacturer’s instructions. Percent cytotoxicity was calculated by normalizing to PBS-treated cells (0% cell death) and Triton X-100-treated cells (100% cell death), as described (Whidbey et al., 2013, 2015b; Boldenow et al., 2016).

### Phyletic Analysis

Phyletic pattern searches were done using the PSI-BLAST program (Altschul et al., 1997) against a locally constructed compressed non-redundant database (NR50) of the National Center for Biotechnology Information (NCBI). Multiple sequence alignments were built using the Kalign program (Lassmann et al., 2009). Contextual information from prokaryotic gene neighborhoods was retrieved by a Perl custom script that extracts the upstream and downstream genes of the query gene. Phylogenetic analysis was conducted using an approximately maximum-likelihood method implemented in the FastTree 2.1 program under default parameters (Price et al., 2010)^1^. Network analysis was performed using the R language with the igraph package (Csardi and Nepusz, 2006).

### Statistical Analysis

A value of *p* < 0.05 was considered significant. Non-linear regression analysis was used to interpolate EC_50_ concentrations. In LDH release assays, an unpaired *t*-test or one-way ANOVA with Tukey’s post-test was employed as appropriate to analyze differences between treatment groups, unless otherwise noted. GraphPad Prism (version 7.03) was used to compute all statistical tests.

## Data Availability Statement

The raw data supporting the conclusion of this article will be made available by the authors, without undue reservation, to any qualified researcher.

## Ethics Statement

The studies involving human participants were reviewed and approved by the Seattle Children’s Research Institute Institutional Review Board (protocol #11117). The patients/participants provided their written informed consent to participate in this study.

## Author Contributions

BA, CW, LI, LA, JC, and LR designed the experiments and analyzed the results. BA, CW, LI, PH-F, PQ, and AH performed the experiments. BA, LI, LA, HJ, and LR wrote the manuscript.

## Conflict of Interest

The authors declare that the research was conducted in the absence of any commercial or financial relationships that could be construed as a potential conflict of interest.

## Footnotes

1 http://www.microbesonline.org/fasttree/
