# Corrigendum: The *cyl* Genes Reveal the Biosynthetic and Evolutionary Origins of the Group B *Streptococcus* Hemolytic Lipid, Granadaene

**DOI:** 10.3389/fmicb.2020.00325

**Published:** 2020-02-25

**Authors:** Blair Armistead, Christopher Whidbey, Lakshminarayan M. Iyer, Pilar Herrero-Foncubierta, Phoenicia Quach, Ali Haidour, L. Aravind, Juan Manuel Cuerva, Heather B. Jaspan, Lakshmi Rajagopal

**Affiliations:** ^1^Department of Global Health, University of Washington, Seattle, WA, United States; ^2^Center for Global Infectious Disease Research, Seattle Children's Research Institute, Seattle, WA, United States; ^3^Computational Biology Branch, National Center for Biotechnology Information, National Institutes of Health, Bethesda, MD, United States; ^4^Department of Organic Chemistry, University of Granada, Granada, Spain; ^5^Department of Pediatrics, University of Washington School of Medicine, Seattle, WA, United States; ^6^Department of Pathology, Institute of Infectious Disease and Molecular Medicine, University of Cape Town, Cape Town, South Africa

**Keywords:** Group B *Streptococcus*, bacterial toxin, microbial evolution, virulence factor, Gram-positive bacteria

There is an error in the Funding statement. The correct numbers for National Institutes of Health are R01AI112619, R01AI133976, R01AI100989, R21AI125907, and T32AI007509.

The authors apologize for this error and state that this does not change the scientific conclusions of the article in any way. The original article has been updated.

