# Supplementary material for: The cyl Genes Reveal the Biosynthetic and Evolutionary Origins of the Group B Streptococcus Hemolytic Lipid, Granadaene
Source: Front Microbiol. 2020 Jan 21;10:3123. doi: 10.3389/fmicb.2019.03123 (PMC6985545; doi:10.3389/fmicb.2019.03123)
Supplement: Supplementary file 1 [file Data_Sheet_1.docx]

Supplementary Material

## Supplementary Figures

**
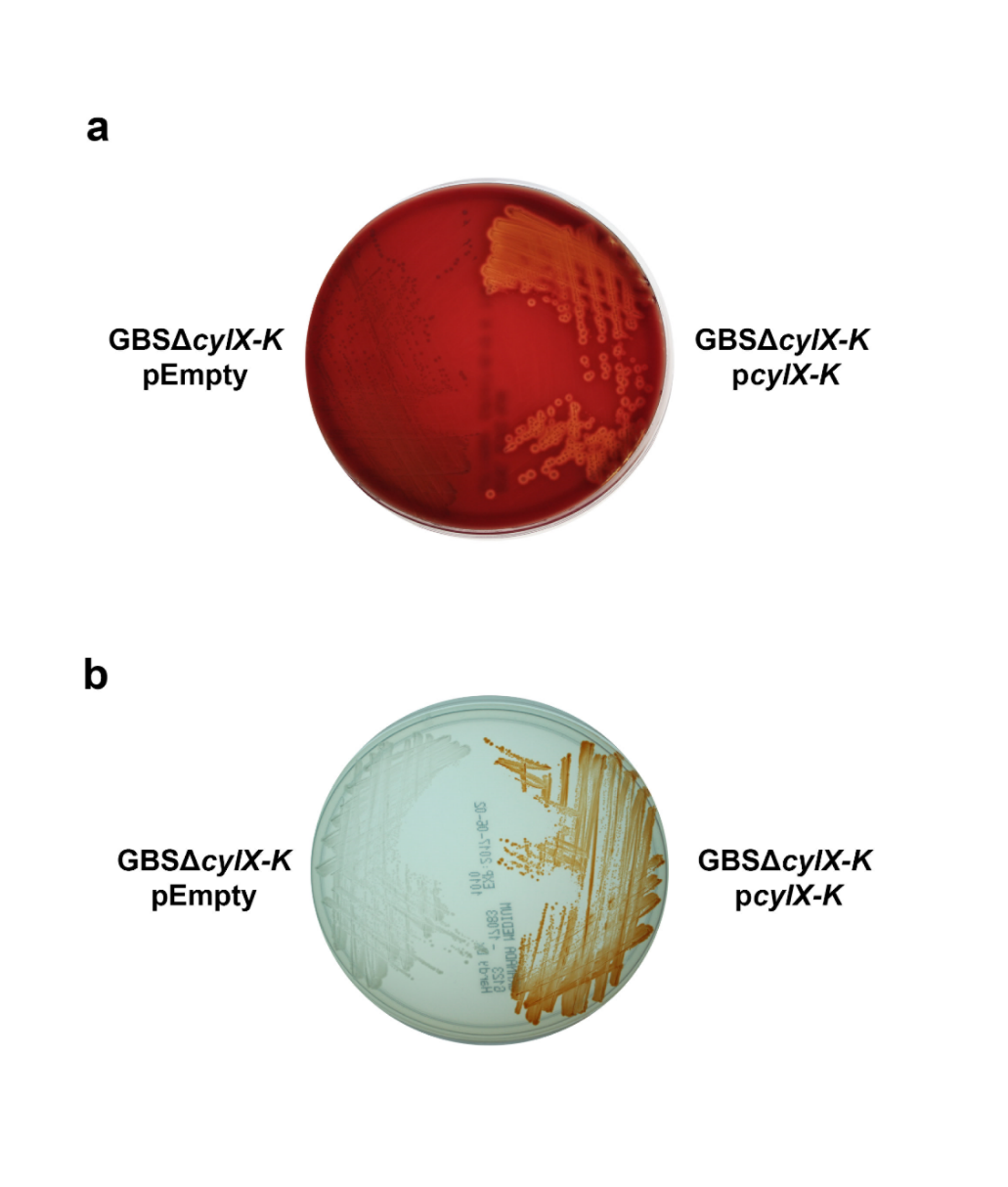
**

**Supplementary Figure 1.** Complementation of the non-pigmented/non-hemolytic GBS strain lacking the *cyl* operon (GBSΔ*cylX-K*(8)) with the p*cylX-K* plasmid, but not the empty plasmid vector (pEmpty), restored hemolysis and pigmentation, as observed on red blood agar (a) and Granada agar (b).


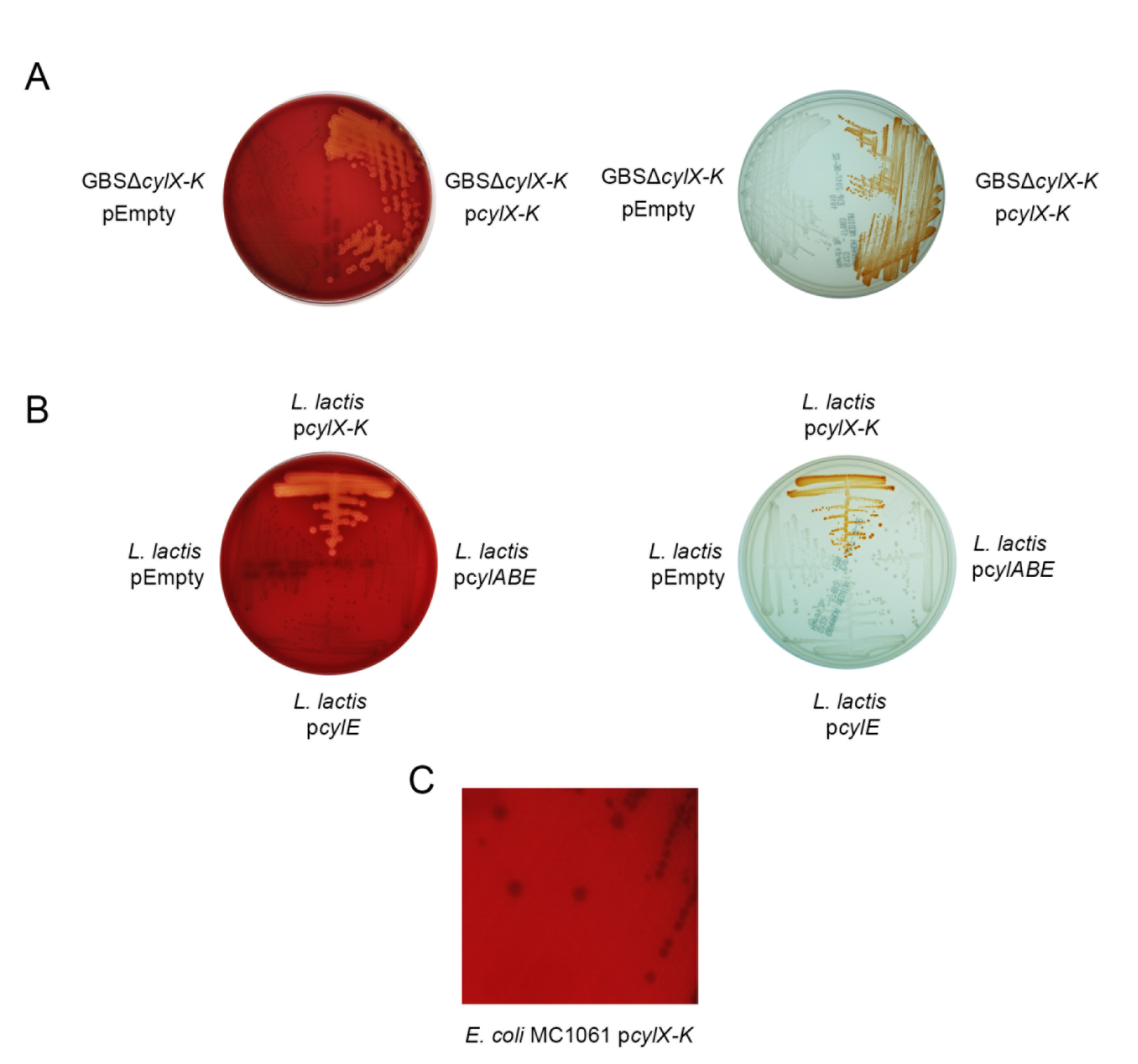


**Supplementary Figure 2.** Introduction of p*cylX-K* into E. coli MC1061 did not confer hemolysis.

**Supplementary Figure 3.** The complete phylogenetic tree of the CylE protein. Clades with strong bootstrap support were collapsed into the filled triangles seen in Fig. 3.


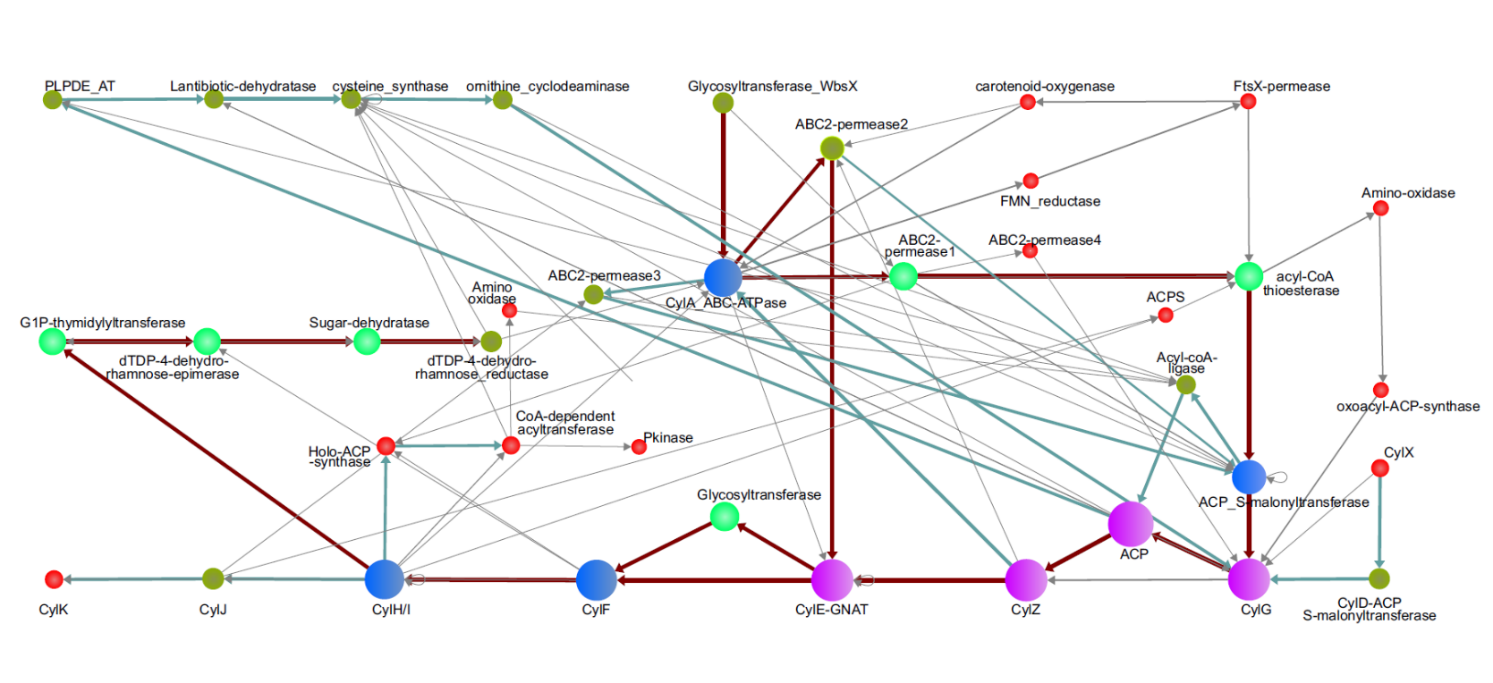


**Supplementary Figure 4.** The operon network of *cyl* genes. Genes linked in operons are connected by arrows with the arrow-head pointing to the adjacent 3’ gene. Node size and edge thickness are scaled based on the number of occurrences.





**Supplementary Figure 5.** Representative chromatogram during HPLC purification of GBS pigment, granadaene. Fractions were collected from 10.5 minutes and 17.5 minutes of each HPLC run (represented by the shaded area) for samples from pigmented or nonpigmented GBS and *L. lactis*.

**Supplementary Tables**

| **Gene** | **Rare codons** | **Frequency** |
| --- | --- | --- |
| *cylX* | Arginine (CGG, AGG) | 4 |
|  | Glycine (GGA, GGG) | 5 |
|  | Isoleucine (AUA) | 2 |
|  | Leucine (CUA) | 2 |
|  | Threonine (ACG) | 2 |
| *cylD* | Arginine (AGG, AGA) | 4 |
|  | Glycine (GGA, GGG) | 5 |
|  | Isoleucine (AUA) | 6 |
|  | Leucine (CUA) | 3 |
| *cylG* | Arginine (AGG, AGA) | 6 |
|  | Glycine (GGA, GGG) | 11 |
|  | Isoleucine (AUA) | 5 |
|  | Leucine (CUA) | 4 |
|  | Threonine (ACG) | 2 |
| *acpC* | Arginine (AGA) | 1 |
|  | Glycine (GGA, GGG) | 2 |
|  | Isoleucine (AUA) | 5 |
|  | Leucine (CUA) | 4 |
|  | Threonine (ACG) | 1 |
| *cylZ* | Arginine (AGA) | 1 |
|  | Glycine (GGA) | 1 |
|  | Isoleucine (AUA) | 3 |
|  | Leucine (CUA) | 2 |
|  | Proline (CCC) | 1 |
| *cylA* | Arginine (CGA, AGG, AGA) | 8 |
|  | Glycine (GGA) | 9 |
|  | Isoleucine (AUA) | 4 |
|  | Leucine (CUA) | 6 |
|  | Threonine (ACG) | 4 |
| *cylB* | Arginine (CGA, CGG, AGG, AGA) | 4 |
|  | Glycine (GGA, GGG) | 7 |
|  | Isoleucine (AUA) | 3 |
|  | Leucine (CUA) | 2 |
|  | Threonine (ACG) | 4 |
| *cylE* | Arginine (CGA, CGG, AGG, AGA) | 25 |
|  | Glycine (GGA, GGG) | 7 |
|  | Isoleucine (AUA) | 11 |
|  | Leucine (CUA) | 5 |
|  | Proline (CCC) | 3 |
|  | Threonine (ACG) | 3 |
| *cylF* | Arginine (AGG, AGA) | 7 |
|  | Glycine (GGA, GGG) | 9 |
|  | Isoleucine (AUA) | 3 |
|  | Leucine (CUA) | 3 |
|  | Proline (CCC) | 1 |
|  | Threonine (ACG) | 1 |

**Supplementary Table S1.** Rare codons are abundant in the GBS *cyl* operon. The sequence of each gene in the *cyl* operon was analyzed using the rare codon sequence analysis tool at <http://people.mbi.ucla.edu/sumchan/caltor.html>. The frequency of each rare codon is listed.

| **Name** | **Sequence** | **T_m_ (°C)** |
| --- | --- | --- |
| cyl1_fwd | 5’ – CGCTAGGAGGAAACAAGGAAGAAGGTGATAATATGGGACG | 79.1 |
| cyl1_rev | 5’ – AAATAGTTAAATAATCCATAGCTGAATACTCTAGC | 63.7 |
| cyl2_fwd | 5’ – CTATGGATTATTTAACTATTTTAGGGATCGTTTC | 65.6 |
| cyl2_rev | 5’ – TTCCAGAAGAATAGGGCTGACATGCCAT | 73.8 |
| cyl3_fwd | 5’ – TCAGCCCTATTCTTCTGGAAAAGGAATCAATTTG | 74.5 |
| cyl3_rev | 5’ – CTTCATTTTGTTATTTACCAGTAGGAACAACATGTTGCG | 74.7 |
| pDC_fwd | 5’ – TGGTAAATAACAAAATGAAGAAAAAG | 60.2 |
| pDC_rev | 5’ – TTCCTTGTTTCCTCCTAGC | 58.5 |

**Supplementary Table S2.** The primers listed above were used to amplify the GBS *cyl* operon for ligation into the multiple cloning site of the pDC123 (pEmpty) plasmid vector using Gibson Assembly. All primers were obtained from Sigma Aldrich.
